# Edge Computing in Nature: Minimal pre-processing of multi-muscle ensembles of spindle signals improves discriminability of limb movements

**DOI:** 10.3389/fphys.2023.1183492

**Published:** 2023-06-29

**Authors:** Jasmine A. Berry, Ali Marjaninejad, Francisco J. Valero-Cuevas

**Affiliations:** ^1^ Brain-Body Dynamics Lab, Department of Computer Science, University of Southern California, Los Angeles, CA, United States; ^2^ Department of Biomedical Engineering, University of Southern California, Los Angeles, CA, United States; ^3^ Division of Biokinesiology and Physical Therapy, University of Southern California, Los Angeles, CA, United States

**Keywords:** muscle spindle afferent, proprioception, limb movement, task discrimination, dimensionality reduction, musculotendon

## Abstract

Multiple proprioceptive signals, like those from muscle spindles, are thought to enable robust estimates of body configuration. Yet, it remains unknown whether spindle signals suffice to discriminate limb movements. Here, a simulated 4-musculotendon, 2-joint planar limb model produced repeated cycles of five end-point trajectories in forward and reverse directions, which generated spindle Ia and II afferent signals (proprioceptors for velocity and length, respectively) from each musculotendon. We find that cross-correlation of the 8D time series of raw firing rates (four Ia, four II) cannot discriminate among most movement pairs (∼ 29% accuracy). However, projecting these signals onto their 1^*st*^ and 2^*nd*^ principal components greatly improves discriminability of movement pairs (82% accuracy). We conclude that high-dimensional ensembles of muscle proprioceptors can discriminate among limb movements—but only after dimensionality reduction. This may explain the pre-processing of some afferent signals before arriving at the somatosensory cortex, such as processing of cutaneous signals at the cat’s cuneate nucleus.

## 1 Introduction

Physical behavior in vertebrates is made possible by hierarchical neuronal systems that send motor commands from the central nervous system to muscles on the basis of peripheral mechanoreceptors that encode musculoskeletal information (proprioceptors for muscle lengths and velocities, tendon forces, joint angles) and haptic skin information. Motor function has received much attention given the relative ease with which the activity of *α* − motoneurons and muscles can be measured and associated with physical behavior. In contrast, the emergence of somatosensory ‘percepts’ (i.e., the transformation from spike trains from an ensemble of mechanoreceptors to a neural impression useful to the control of movement) has proven much more challenging to understand. This is because the actionpotentials from mechanoreceptors on the skin, muscles and joints are not easily isolated or recorded (Tabot et al., 2013), and the somatosensory percepts they elicit in the central nervous system cannot be readily measured.

The lack of understanding of the transformation from proprioceptive signals to somatosensory percepts is particularly problematic to the study and theories of sensorimotor control (Loeb et al., 1990). In particular, somatosensory percepts (also called kinaesthesia) provide the sense of self-movement and body configuration and position (Ferrell et al., 1987). In addition, it is only via the parallel processing of raw sensory signals at cortical and subcortical levels that it is possible to detect salient features of the body that can appropriate motor actions to be selected, elicited and implemented (Cisek and Kalaska, 2010; Berry and Valero-Cuevas, 2020). Rigorous neurophysiological work on mechanoreceptors shows that Ia and II muscle spindle afferents preferentially encode the rate of change of muscle fiber length, and muscle fiber length, respectively (Mileusnic et al., 2006). The fact that muscle fiber lengths and velocities are related to the angles and angular velocities of the joints their musculotendons cross, respectively, Valero-Cuevas (2016) has led to the assumed fundamental tenet of sensorimotor control that muscle spindles provide limb configuration information for adaptable, accurate, and robust control of limb movement.

Recent computational work suggest that spindle information is likely necessary to extract a percept of body configuration (Berry et al., 2017; Berry and Valero-Cuevas, 2020; Sandbrink et al., 2020), but not sufficient as it may need to be conditioned on tendon tension information from Golgi tendon organs (Hagen et al., 2021). Notwithstanding the geometrically obligatory relationship between joint angles and musculotendon lengths, the tenet that spindle information is a primary source of body configuration (even when conditioned on Golgi tendon organ signals) remains to be proven as musculotendons often span multiple joints (Valero-Cuevas, 2016) and spindle signals can be modulated independently of joint angles by *γ* − motoneuron drive to their intrafusal fibers in which the mechanoreceptors sit (Jalaleddini et al. (2017) and references therein). Moreover, this tenet cannot be demonstrated experimentally because spindle afferent recordings from numerous limb muscles in peripheral nerves or dorsal root ganglia cannot be measured directly during large limb movements. In spite of the lack of conclusive evidence for this tenet, it is regularly adopted to the point that other mechanoreceptors also affected by joint angles (i.e., synovial capsule, ligaments and skin) and Golgi tendon organs (which respond to tendon force) are considered secondary for the control of movement for reasons detailed in the Discussion.

The main motivation for our computational approach based on principal components analysis (PCA) is the inability to empirically record large ensembles of spindle afferent signals during movement, and the evidence that pre-processing of tactile information is analogous with PCA (Sanger, 1989; Rongala et al., 2018). In particular, we performed a computational experiment to assess the ability of raw *versus* pre-processed spindle afferent signals to provide usable limb configuration information. Such pre-processing has been described for tactile signals in the cuneate nucleus in the brainstem of the cat (Rongala et al., 2018). This subcortical pre-processing is an example of parallel processing advocated for biological systems (Cisek and Kalaska, 2010). Mearns et al. (2020) used high-speed videography to investigate the hunting behavior of larval zebrafish, focusing on the neural circuits and mechanisms involved in the behavior. Findings showed how hunting behavior is mediated by a tightly coupled stimulus-response loop, with distinct populations of neurons controlling different aspects of the behavior. Additionally, Marques et al. (2018) used unsupervised clustering algorithms to classify the various types of locomotor behavior exhibited by zebrafish larvae, consequently finding that the zebrafish locomotor repertoire consists of a small number of discrete behavioral states, each of which is associated with a distinct set of motor patterns. Several genetic mutations were identified that altered the zebrafish locomotor repertoire, suggesting that the genetic basis of behavior is closely linked to the neural circuits that control it. Unlike centralized and serial engineering controllers, animals tend to have a distributed and hierarchical neural structure for the processing of motor and sensory signals (Grillner and Wallen, 1985; Schwab and Coates, 2003). Such peripheral pre-processing of the flood of somatosensory information is analogous to the use of parallelized ‘Edge Computing,’ which affords temporal and computational efficiencies when massive amounts of data from different sources are involved (Varghese et al., 2016).

As in Rongala et al. (2018), we use the statistical notion of discriminability (i.e., distinguish task A from B) as a minimal requirement for the utility of sensory percepts. Discriminability has also been used to test how raw and processed signals from skin mechanoreceptors on the fingertips can be used to distinguish among different edges and textures to inform manipulation (Saal et al., 2017; Okorokova et al., 2019). In this investigation, we tested the extent to which raw *versus* pre-processed ensembles of Ia and II spindle afferents signals could discriminate among the five simulated limb movements that produced them.

## 2 Materials and methods

The computational design of the simulated tendon-driven system, the trajectories selected for inspection, and the modified spindle afferent model is detailed further in this section. Then we’ll detail the methods of pre-processing and filtering used to reduce the dimensions of afferent signals. Our pre-planned trajectories produced afferent signals that were compared in inter-class contexts in then processed in data series estimation, pattern identification, and unsupervised machine learning algorithms on the resulting afferents to reveal their spatial and temporal dynamics. Lastly, we’ll conclude with a review of how the feature selection and extraction techniques were implemented to determine which relevant spindle model features maintained substantial effects in classifying one trajectory from another within sensory space.

### 2.1 Kinematic model structure and parameters

**FIGURE 1:**
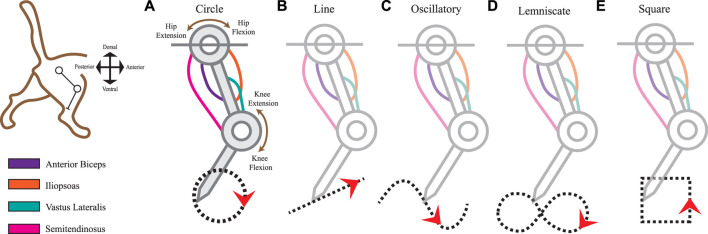
Limb kinematics were derived from distinct leg movements that produced different ecological end-point trajectories. The 2-joint planar kinematic limb model has four muscles analogous to those found in the cat hindlimb: anterior biceps, iliopsoas, vastus lateralis, and semitendinosus. We used the model to produce five endpoint trajectories The red arrowheads indicate the default movement direction (also studied in the reverse direction). **(A)** Circle trajectory in a clockwise direction. **(B)** Line (point-to-point) trajectory from left to right. **(C)** Oscillatory (sinusoidal) trajectory from left to right. **(D)** Lemniscate (“figure-eight”) trajectory. **(E)** Square trajectory in a counterclockwise direction.

We constructed a simplified tendon-driven leg model, represented as the feline hindlimb, with a pivot at the hip joint. In tendon-driven anatomies, tendons are responsible for permitting muscles to act on vertebrate limbs and actuating the kinematic Degrees of Freedom (DOF) (Valero-Cuevas and Santello, 2017). The planar model consisted of four muscles, two links, and two DOFs (Hip Flexion/Extension and Knee Flexion/Extension) connecting the thigh and shank, as shown in Figure 1. For simplicity we excluded actuation of the foot (i.e., paw), which is normally included in a feline hindlimb model and would be more representative of the actual feline. Lengths of the thigh and shank segments were set to 90 mm and 100 mm, respectively, with musculature comparable to the muscle-joint interactions and parameter data resulting from system identification analyses (Harischandra and Ekeberg, 2008) that were based on mathematical properties of skeletal muscle formulated by Zajac (1989).

To imitate the useful dynamics of the cat’s hindlimb mobility, we captured the movements of the leg as generated by 4 muscles: *Anterior Biceps (AB)*, *Iliopsoas (IL)*, *Vastus Lateralis (VL)*, and the *Semitendinosus (SM)*. Table 1 summarizes the parameters we used in the musculo-tendon structure which contained parameters of maximal length as *L*_*max*_, constant moment arm values as *r*, and optimal length values (*L*_*O*_) per muscle at the reference angle. Figure 1 depicts the tendon routing of *AB*, *IL*, and *VL* as unifunctional joint muscles. *AB* and *IL* are acting in paired antagonistic form on the hip. *VL* activates knee extension movements, while *SM* serves as a bifunctional joint muscle acting on both the hip and knee. According to Harischandra and Ekeberg (2008), the resting (neutral) posture of the hip at 65° and the knee at 100° maintained mono-articulated muscles at a length of 85% of *L*_*max*_ and 75% for bi-articulated muscles.

**TABLE 1:** Simulated limb and musculotendon parameters.

Muscle name	*L*_*max*_ (mm)	Angle movement	Moment arm (mm)	Reference angle *L*_*O*_ (%)
*Anterior Biceps*	70	Hip Extension	30	85
*Iliopsoas*	70	Hip Flexion	−44	85
*Vastus Lateralis*	50	Knee Extension	9	85
*Semitendinosus*	70	Hip Extension	30	75
		Knee Flexion	−38	

Optimal lengths (*L*_*O*_) of each muscle at the reference angle were set to 85% and 75% of *L*_*max*_ for unifunctional and bifunctional muscles, respectively.

### 2.2 Trajectory planning

Arbitrary shapes were selected as pre-planned trajectories in two-dimensional planar space for the end-effector limb positions. All trajectories were performed in closed loops and mathematically expressed as parametric functions of time, *t*, to obtain the *x* and *y* coordinate locations. The cat limb executed five point-to-point movements that will be further referred to as *task representations*. Each task representation contained a total of 200 equidistant points per cycle on the trajectory. One full cycle lasted for a time frame of one cycle/second.

The first task representation is the Circle trajectory (Figure 1A) which prompted the limb to perform uniform circular motion within a 5 mm diameter in the clockwise direction. The end-effector’s total distance traveled approximates to 15.71 mm. Next, the Line trajectory (Figure 1B) positioned the end-effector on the path of a straight line to simulate smooth, uninterrupted movement along a ramp. Relative to the horizontal plane, the line segment retained a 50% incline at 26.57° steepness. Its midpoint position was at the 100 mm y-intercept on the Cartesian plane. The total distance traveled for one cycle of the Line trajectory was 12 mm.

The Oscillatory trajectory (Figure 1C) is a sinusoidal wave forming a path of a smooth periodic oscillation. Using Eq. 1 as a function of time, the amplitude *A* was set to 20 mm with a frequency, *f*, of 10 Hz. The angular frequency, *w*, expressed in radians at run-time Eq. 2 along with zero phase shift, *φ*.

ω=2πf (1)

$$y_{n}\left(t\right)=A\sin\left(\omega t+\varphi \right)$$ (2)

The Lemniscate trajectory (Figure 1D) created two symmetrical and uniform-sized lobes to form a shape resembling the “figure-of-eight” curve (Richardson and Flash, 2002). The curve was formed using parametric curves from Eq. 3 and Eq. 4.

$$x_{n}=4\times 10^{-2}\sin\left(5\times 10^{-1}t\right)$$ (3)

$$y_{n}=2\times 10^{-1}\sin\left(t\right)+11\times 10^{-2}$$ (4)

Lastly, we prescribed the Square trajectory (Figure 1E) as a proximity comparison to the Circle trajectory movement. Considering squares and circles are topologically equivalent shapes, we expected to view closer spatiotemporal similarities in the sensory space between these two shapes over others. However, squares differ in their non-continuity and finite lines of reflectional symmetry which also might reflect symmetry within the afferent manifolds. To what extent will the afferent signals reflect these features in the observed kinematics and make the muscle activities and joint motions distinguishable is one facet of the experimental outcomes we sought to observe.

The limb joints on the planar limb actuate as revolute joints with links capable of rotating around it. The 2 links comprise of an end effector which maintains the foot position at the end of the shank link and also the end of the articulated body. While the hip position remained affixed as the root joint, we calculated the tracing of the end effector position across each of the five trajectories using inverse kinematics. For each trajectory, the 200 target positions in the Cartesian space were selected as inputs for the inverse kinematics algorithm and the limb pose (i.e., state) required for the target position were derived to determine the joint angles at the hip and knee, *q*_1_ and *q*_2_ respectively.

Inverse kinematic solutions are generally not unique, and are sometimes dependent on the initial joint coordinated *q*_0_, which typically defaults to value 0. However, the *θ* values for *q*_1_ and *q*_2_ of the limb were successfully obtained despite the possibility of a multiplicity of joint angles producing the same end-effector position. Given the desired limb’s end-effector positions, for each time step across the trajectory at instance *i*, the segment link lengths, *l*_1_ and *l*_2_, and the coordinate positions, *x*_1_ and *x*_2_, were recorded to calculate variables *c* and *s* in Eq. 5 and Eq. 6, respectively. Joint angles *q*_1_ and *q*_2_ for each segment were then iteratively derived using equations Eq.7 and Eq.8.

$$c=\frac{\left(x_{i}^{2}+y_{i}^{2}-l_{1}^{2}-l_{2}^{2}\right)}{\left(2l_{1}l_{2}\right)}$$ (5)

$$s=\sqrt{1-c^{2}}$$ (6)

$$q_{1}=\sin^{-1}\frac{y_{i}\left(l_{1}+l_{2}c\right)-x_{i}l_{2}s}{x_{i}^{2}+y_{i}^{2}}$$ (7)

$$q_{2}=\cos^{-1}\frac{x_{i}^{2}+y_{i}^{2}-l_{1}^{2}-l_{2}^{2}}{\left(2l_{1}l_{2}\right)}$$ (8)

Once the limb’s joint angles are calculated, a Jacobian matrix can be generated to determine the relationship between simulated limb’s joint parameters and the end-effector velocities. The change in joint angles are then used as inputs for the muscle spindle model to obtain raw sensory afferents for each trajectory.

### 2.3 Muscle spindle afferent data collection

In a similar method that was used in Berry et al. (2017), the joint and limb kinematics were solved using a computational sub-model to simulate the biological spindle as observed in mammalian muscles, namely that of the cat (Mileusnic et al., 2006; Mileusnic and Loeb, 2006), which has also been used in human simulations (Song et al., 2008; Laine et al., 2016). Action potentials in pulses per second (pps) were generated for primary (Ia) and secondary (II) afferents based on the interactions of the intrafusal fibers (chain, bag1, bag2). Fusimotor activation and the property changes in induces within the spindle model is represented by contractile elements (CE). The spindle model operates from a set of parameterized inputs that included *L*_*o*_ as optimal muscle lengths, *L*_*ce*_ as muscle length normalized to *L*_*o*_, *V*_*ce*_ as the rate of change in muscle length (i.e., velocities), *A*_*ce*_ as muscle length acceleration, *Fs* as sampling frequency, *γ*_*dynamic*_ as dynamic gamma drive, and *γ*_*static*_ as static gamma drive.

The model produced only two outputs, which were non-linear firings of the primary afferent potential and secondary afferent potential modalities in the spindle, Ia and II respectively. As stated in Mileusnic et al. (2006), the generation of afferent potential reflects the stretch of the intrafusal fiber model’s sensory zone. Afferent potential primary is derived based on Eq. 9 where *T*/*K* ^*SR*^ is the calculated stretch in the sensory region of each intrafusal fiber, $L_{N}^{SR}$ is the sensory region threshold length, $L_{0}^{SR}$ is the sensory region rest length, and *G* is a constant that indicates the numerical relationship between intrafusal fiber’s sensory region to primary afferent firing. Afferent potential secondary derived based on Eq. 10 where *X* is the percentage of the secondary afferent located on the sensory region and *L*_*secondary*_ is the secondary rest length.

$$Ia\;Afferent\;Potential=G\cdot \left[\frac{T}{K^{SR}}-\left(L_{N}^{SR}-L_{0}^{SR}\right)\right]$$ (9)

$$\begin{array}{ll} & II\;Afferent\;Potential\\ & \;=G\cdot \left\{X\cdot \frac{L_{\mathrm{secondary}}}{L_{0}^{SR}}\cdot \left[\frac{T}{K^{SR}}-\left(L_{N}^{SR}-L_{N}^{0}\right)\right]\right.\\ & \;\;\left.+\left(1-X\right)\cdot \frac{L_{\mathrm{secondary}}}{L_{0}^{PR}}\cdot \left(L-\frac{T}{K^{SR}}-L_{0}^{SR}-L_{N}^{PR}\right)\right\} \end{array}$$ (10)

Both of the afferent firing model’s output firings were collected as raw data to be statistically analyzed for useful features that would indicate the current state of the limb.

### 2.4 Comparison of inter-class trajectory context

In order to evaluate the discriminability of afferent signals against task-actions, the trajectory types must be compared extensively. The five trajectories selected for inspection are cycles of shapes and curvatures that are not typically associated with the natural gait of a feline hind limb: Circle, Line, Oscillatory, Lemniscate, and Square. For this reason, there is an increased likelihood to indisputably discern variations despite noise that may be present with a data set’s dimensionality, resolution, and sparsity. In our initial simulation executions, we observed that sensory afferent outputs of the muscles varied significantly depending on the initial conditions and the direction the limb moves in to complete the cycle. Therefore, we ensured that the simulated limb traversed each of the trajectories in two opposite directions: Reverse (REV) and Forward (FWD). For example the Circle-FWD, which indicates the limb traversed the Circle trajectory moving in the Forward direction, was compared in series to Circle-REV, Line-FWD, Line-REV, Oscillatory-FWD, Oscillatory-REV, Lemniscate-FWD, Lemniscate-REV, Square-FWD, and Square-REV. All possible combinations of trajectory comparisons totaled to 45 correlation pairs in both the raw data set and pre-processed (i.e., PCA) data set. The combination set did not include pairs that evaluated a trajectory-direction against each other.

### 2.5 Spatial, spatio-temporal, pre-processing of muscle spindle afferent data

To test the presence of discriminability across tasks, the afferent data sets were evaluated within 3 pattern constraints: spatial, spatio-temporal, and pre-processing from dimensional reduction.

#### 2.5.1 Spatial analysis

We first evaluated the spatial patterns using the K-means++ algorithm. Since the standard K-means algorithm does not guarantee to find the optimum, an alternative, K-means++ chooses initial centers on a justifiable upper bound within a cluster sum of squares objective. The approach is initiated by separating the *k* initial cluster centers, spatially.

Overall the formal objective is to determine:

$$\underset{S}{\arg\;\min}\overset{k}{\underset{i=1}{\sum }}\underset{x\in S_{i}}{\sum }{\left\|x-\mu_{i}\right\|}^{2}=\underset{S}{\arg\;\min}\overset{k}{\underset{i=1}{\sum }}|S_{i}|\;\mathrm{Var}\;S_{i}$$ (11)

where *μ*_*i*_ is the mean of points in *S*_*i*_. This may also be shown to be equivalent to minimization of the squared deviations of points, as shown by:

$$\underset{S}{\arg\;\min}\overset{k}{\underset{i=1}{\sum }}\;\frac{1}{2|S_{i}|}\;\underset{x,y\in S_{i}}{\sum }{\left\|x-y\right\|}^{2}$$ (12)

For an initial set of *k* means $m_{1}^{\left(1\right)},\ldots ,m_{k}^{\left(1\right)}$ , the algorithm proceeds by alternating between the assignment step and an update step, until convergence.

#### 2.5.2 Spatio-temporal analysis

A useful statistical measure to use that identifies significant correlations among multiple trajectories with spatial and temporal components is cross correlation. It compares the time-series of afferent data across tasks, and is represented as the ratio in Eq. 13, where *n* is the total number of data point indices recorded per task cycle. This is suitable for measuring well two variables move in relation to each other. Both *x*_*i*_ and *y*_*i*_ are the individual spindle afferent sets, Ia and II, respectively. A temporal shift delay, phase lag *τ*, of the output cross correlation, *R*_*xy*_, measure is applied to determine where the correlation of the data is maximized, as shown in Eq. 14

$$R_{xy}\left(\tau \right)=\frac{\sum_{i=1}^{n}\left(x_{i}-\bar{x}\right)\left(y_{i}-\bar{y}\right)}{\sqrt{\sum_{i=1}^{n}{\left(x_{i}-\bar{x}\right)}^{2}\sum_{i=1}^{n}{\left(y_{i}-\bar{y}\right)}^{2}}}$$ (13)

$$\tau_{\mathrm{estimated}}=\underset{\tau \in R}{\arg\;\max}\left(R_{xy}\left(\tau \right)\right)$$ (14)

To retrieve the correlation coefficients, local sums can be calculated in an alternative way to normalize the cross-correlation. Using normalized cross correlation follows a general procedure by Lewis (2005) and Haralick and Shapiro (1992) in Eq. 15

$$\gamma \left(u,v\right)=\frac{\sum_{x,y}\left(f\left(x,y\right)-{\bar{f}}_{u,v}\right)\left(t\left(x-u,y-v\right)-\bar{t}\right)}{\sqrt{\sum_{x,y}{\left[f\left(x,y\right)-{\bar{f}}_{u,v}\right]}^{2}\sum_{x,y}{\left[t\left(x-u,y-v\right)-\bar{t}\right]}^{2}}}$$ (15)

We treat the combined group of muscle modalities within the afferent data as an image and template correlation and calculating the cross-correlation in the spatial or the frequency domain. The implementation closely follows the formula from Lewis (2005), where *f* is the image, $\bar{t}$ is the mean of the template, and ${\bar{f}}_{u,v}$ is the mean of *f*(*x*, *y*) in the region under the template.

#### 2.5.3 Data pre-processing for principal component analysis

We investigated the ability of muscle proprioceptors to discriminate among different limb movements, specifically focusing on spindle Ia and II afferent signals. This approach to percepts is inspired by how tactile signals are able to discriminate among touch inputs in Rongala et al. (2018). Initially, the raw firing rates of four Ia and four II afferent signals were cross-correlated in an 8-dimensional time series. However, such cross-correlations in the native high-dimensional space did so poorly (see Results in Section 3). Therefore, we opted to apply cross-correlation after dimensionality reduction via principal component analysis (PCA). Principal component analysis is a method of dimensionality reduction often used to 1) increase computational efficiency: with fewer features, machine learning algorithms can run faster and require less memory, 2) remove noise and redundancy from data, 3) improving the accuracy of subsequent analyses, and 4) visualize data in lower dimensions: making it easier to see patterns and relationships among variables.

In this case, we use PCA as a dimensionality-reduction method to identify the principal components that represent the most variance between the Ia and II signals. This is an extension of our work that explores the use of PCA as a tool to understand and characterize the degrees of freedom of neuromechanical systems Kutch and Valero-Cuevas (2012); Valero-Cuevas (2016); Clewley et al. (2008); Bartsch-Jimenez et al. (2023). In those works, we describe the advantages and limitations of PCA in detail. Briefly, its limitations include its assumption of orthogonality and linearity of the bases found, loss of information about subtle features, sensitivity to outliers and variable scaling, requirement of large data sets, assumption of unimodal distributions of data, and unsuitability for categorical data. Nevertheless, PCA can be a very powerful tool to identify the dominant low-dimensional structure of high-dimensional ensembles of physiological signals.

Algorithm 1 Principal Component Analysis (PCA).

 1: **Input**: Form row vectors **x**_*i*_ = (**x**_1*i*_, **x**_2*i*_, …, **x**_*di*_) from *n* data points. Data matrix $X\in R^{n\times d}$ , size *n* × *d*, number of components *k*
 2: **Output**: Matrix of transformed data $Z\in R^{n\times k}$

 3: **Procedure**: Compute the mean of each variable $\bar{x}=\frac{1}{n}\sum_{i=1}^{n}x_{i}$

 4: Subtract the mean of each column from matrix **X** to center the data
 5: Calculate the covariance matrix $C=\frac{1}{n}X^{T}X$

 6: Compute the eigenvectors and eigenvalues of **C**: **CV** = *λ* **V**, where $V\in R^{d\times d}$ and $\lambda \in R^{d}$

 7: Sort the eigenvalues in descending order and select the *k* largest eigenvectors corresponding to the *k* largest eigenvalues to form the matrix $U\in R^{d\times k}$

 8: Transform the data using **Z** = **XU**
 9: **return reduced** **Z**

As shown in Algorithm 1, PCA functions as an unsupervised learning algorithm that takes a data matrix **X** as input and outputs a matrix of transformed data **Z** of extracted low dimensional feature vectors, where the data have been projected onto the *k* principal components. The algorithm first centers the data by subtracting the mean of each column from **X**. Then, it calculates the covariance matrix **C** of the entire data set. Next, select the top *k* eigenvectors, which value depends on the number of dimensions we want to reduce the data to and that explain the most variance in the data. The eigenvalues are sorted in descending order and the *k* largest eigenvectors are selected to form the matrix **U**. Finally, the data are transformed by computing the dot product **Z** = **XU** and the transformed data samples are returned in a new subspace. This new space consists of the principal components, which are the transformed data points that capture the most significant variation in the original data.

## 3 Results

### 3.1 Raw multi-dimensional afferent signals are bounded, but movement-indiscriminable

**FIGURE 2:**
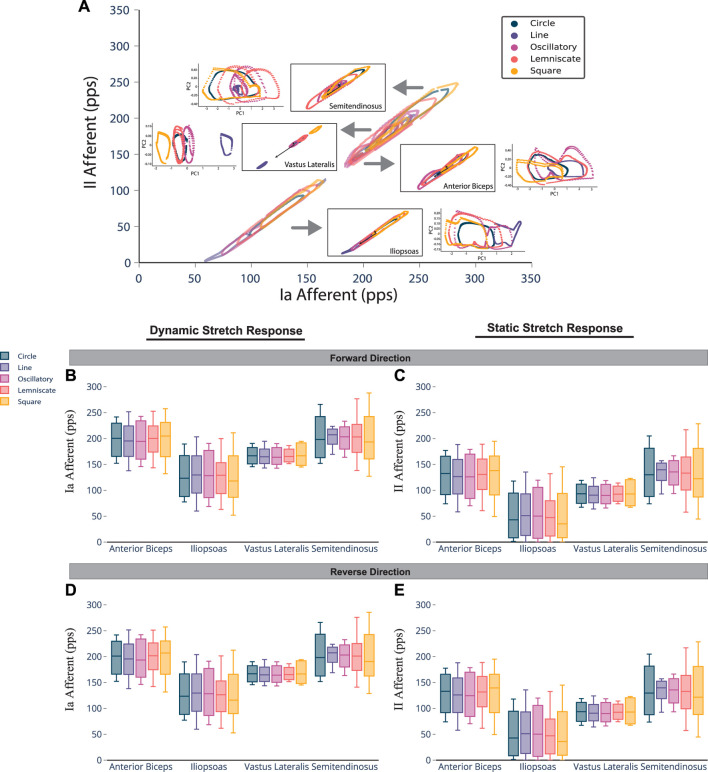
Spindle afferent signals for all end-point trajectories in the forward and reverse directions. Macro-level evaluation of the raw afferent signals reveals that an individual task trajectory and muscle are significantly more difficult to disambiguate from others before pre-processing. However, a micro-level observation of the Ia and II afferent signals shows that a muscle will maintain similar dispersion of values (i.e., maximum score, minimum score, and interquartile ranges) across various tasks, in both forward and reverse directions. [**(A)**, Top Row] Comprehensive view of all raw proprioceptive signals charted and overlaid together (plotted on main axis) and separated by muscle group (boxed subplots). Pre-processed signals for each muscle are shown as PC1 vs. PC2 scatter plots. [**(B)**, Middle Row, Left] Distribution and skewness of Ia afferent signals (fiber velocity, or dynamic stretch response) for each trajectory performed per muscle. [**(C),** Middle Row, Right] Distribution and skewness of II afferent signals (fiber length, or static stretch response). [**(D,E)**, Bottom Row] Same as **(B,C)**, but for end-point trajectories in the reverse direction.

The Ia and II afferent signals from each muscle (in pulses per second, pps) were recorded for 200 points along each end-point trajectory. In Figure 2A, the scatter plot of Ia-II afferents and boxed subplots of individual muscles (Tables 2, 3) show these pre-processed signals are difficult to disambiguate for proper state estimation. However upon closer inspection, the per-muscle, per-type (Ia vs. II), and per-direction boxplots (Figures 2B–E) show the afferents to be uniquely bounded and limited, likely according to the anatomical muscle length. Although the end-point trajectories (in either direction) are indiscriminable on the basis of the ranges of raw Ia and II signals, the observed lower and upper bounds of each respective muscle group demonstrate approximate optimal value ranges to expect afferentation. We then tried clustering as an initial baseline of the ability of unsupervised methods to distinguish among limb trajectories on the basis of these simulated proprioceptive signals. For this, we used the *K*-means++ clustering analysis. Not surprisingly, it only detected K = 3 clusters (not shown), which did not correctly detect the expected 10 overlapping clusters (5 types of end-point trajectories from one another, also in their reverse directions). This served to motivate the alternative approach we developed to successfully disambiguate among limb trajectories on the bases of Ia-II afferent signals.

**TABLE 2:** Ranges of Ia afferent activity, measured in pulses per second (pps), for muscle groups averaged across Forward and Reverse directions.

Muscle name	Maximum (*p*)	Median	Minimum (*q*)
*Anterior Biceps*	249.07	199.4	142.22
*Iliopsoas*	199.33	125.22	64.15
*Vastus Lateralis*	191.18	165.5	145.77
*Semitendinosus*	257.17	200.55	150.50

**TABLE 3:** Ranges of II afferent activity, measured in pulses per second (pps), for muscle groups averaged across Forward and Reverse directions.

Muscle name	Maximum (*p*)	Median	Minimum (*q*)
*Anterior Biceps*	185.66	130.99	62.55
*Iliopsoas*	130.16	45.38	.28
*Vastus Lateralis*	119.855	92.01	67.73
*Semitendinosus*	194.83	132.08	72.53

### 3.2 Pre-processing suggests observable correlations in sensory and motor maps

**FIGURE 3:**
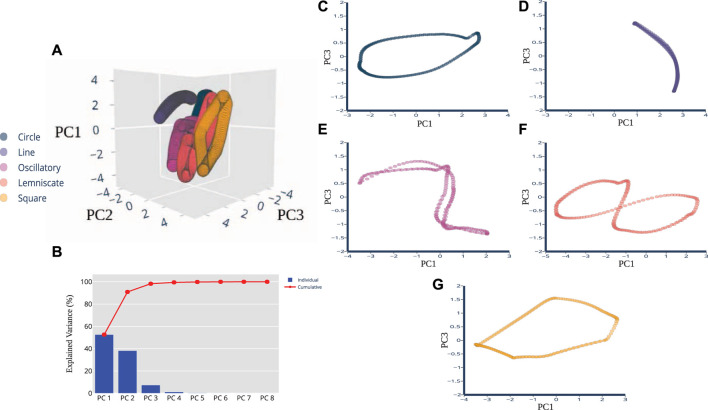
PCA Dimensions of Afferents Reveal Distinct Shapes. Principal component plots of pre-processed data revealed shapes that are quantitatively correlated to the planned trajectory cycles in kinematic space. **(A)** A three-dimensional PCA plot shows the cluster of samples based on their similarity, revealing distinctive shapes in space. **(B)** PCA scree plot of the variance explained by each of the 8 individual principal components are shown here in blue, with cumulative percentages show in red. The first 3 PCs explain 99.61% of the variance. The two-dimensional plots of the **(C)** Circle, **(D)** Line, **(E)** Oscillatory, **(F)** Lemniscate, and **(G)** Square shapes show more distinction in visual appearance of the trajectory when the PC1 and PC3 variables were plotted together.

We used Principal Component Analysis (PCA) as the pre-processing technique because (**i**) certain artificial neural networks can be thought of as performing classical statistical techniques like PCA (Sanger, 1989), and (**ii**) several studies of multidimensional sensory signals demonstrate PCA is a useful means to establish discriminability (Saal et al., 2017; Rongala et al., 2018; Okorokova et al., 2019). After pre-processing, not only did we find strong task-relevant groupings but also shape similarities between the spatiotemporal features of the pre-processed sensory signals and the corresponding end-point trajectory. In Figure 3A, the top 3 principal components are plotted for the five trajectories. The projections overlap each other significantly and are tightly clustered along the same plane. Most of the explained variance (99.03%) is captured in the first 2 principal components. PC1 captures the most variation at 70.22%, PC2 follows with 28.81%, and PC3 captures 0.58%. Figure 3B displays the breakdown for each component with their individual and then the overall cumulative values. When the principal components for each individual trajectory were plotted separate from one another, we were able to perceive discernible shapes that were not visible, but possibly obscured, in the raw data set. In Figures 3C–G, PC1 and PC3 revealed projections that closely resembled the prescribed trajectories and task in the joint kinematic space. Figure 3C, associated with the Line trajectory, reveals a non-straight line with slight curvature. Figure 3E captures the full revolution of the Oscillatory task. One half of the task’s revolution does not completely trace over the other half, unlike the Line, but overall afferent response still reveals the sinusoidal shape. The Square trajectory roughly resembles the planned trajectory, except the sides are not quite equilateral and roughly resembles a parallelogram. Some distortion is acceptable here and not indicative of any errors in the dimensionality reduction of the data. In fact, the results of near-identifiable shapes emerging from the principal components were surprising and not expected, considering the raw data presented clusters of oval-like shapes.

Since the pre-processed data were able to be visualized with 2-3 principal components there was no need to consider other dimension reduction techniques such as T-distributed Stochastic Neighbor Embedding (t-SNE) and multidimensional scaling (MDS). Two or three principal components are usually sufficient for our plotting purposes whereas for classification or modeling purposes, the number of significant components can be properly determined using metrics such as the explained variance. Here, we were able to conclude that there is a presence of near-approximate quantitative correlations of joint kinematic and sensory space of the muscle spindle. The next experimental findings further use these top three components to determine their usability for state classification.

### 3.3 Correlation index reveals markers of action discriminability, classification

**FIGURE 4:**
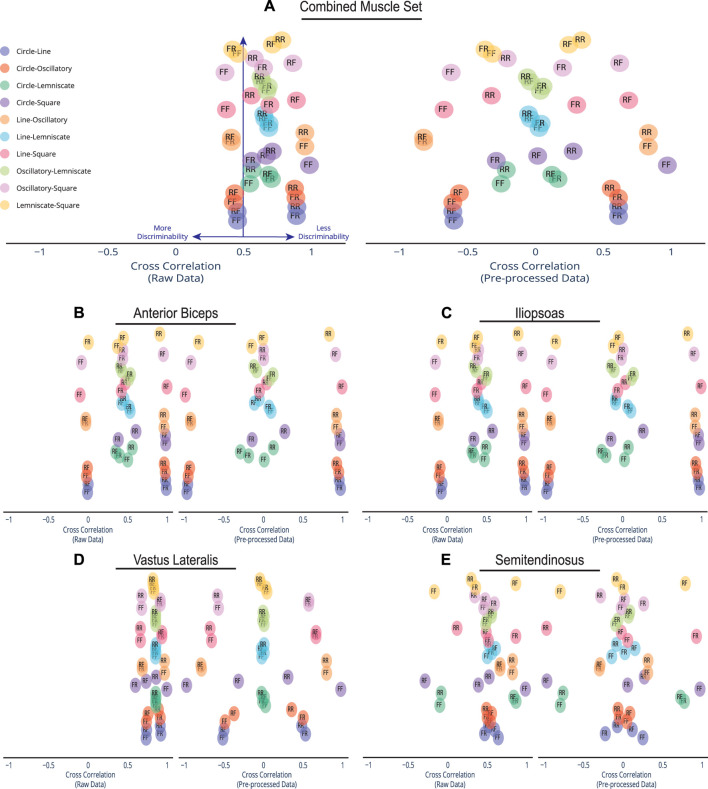
Spread of Discriminability Within Cross Correlation Scatter. Compared to the dense overlap in cross correlations of the raw afferent data for each muscle group, pre-processing spreads out the correlations and improves discriminability. Depicted here are the cross correlations of all possible trajectory combinations (*n* = 45) for the raw information and pre-processed afferent signal information, which are plotted on the left and right, respectively. Each trajectory pairing has an assigned direction. An *R* label is for Reverse Direction and an *F* label is for Forward Direction. For example, the Circle-Line pairing with label *FF* indicates Circle going Forward and Line going Forward. The Line-Square pairing of *RF* indicates Line going in Reverse and Square going Forward. Cross-correlations were plotted for **(A)** the combined set of the 4 muscles in the cat limb, **(B)** Anterior Biceps, **(C)** Iliopsoas, **(D)** Vastus Lateralis, and **(E)** Semitendinosus. Note that preprocessing the data using PCA produces much greater discriminability among movements.

Before pre-processing the afferent manifolds to detect useful features, cross correlation was performed on the raw data set to retrieve the correlation coefficient or index value that measures similarity in movements of two time-series sets of data relative to each other. To our dissatisfaction, cross-correlation analysis, as computed from Eq. 13, did not provide sufficient discriminability among the five states when comparing the raw spindle manifolds. A positive 50%, the measure of chance, was set as the threshold for verifying discriminability among the span of possible cross correlation values where the value −1 indicates a perfect negative correlation, +1 indicates the perfect positive correlation, and 0 is no correlation between the paired tasks. Essentially, *R*_*xy*_(*τ*) ≥ 0.5 indicates less discriminability among the tasks and *R*_*xy*_(*τ*) < 0.5 indicates more discriminability. Assessments for cross correlation were divided into 5 sensory afferent groups: combined muscles set (all four muscles combined), Anterior Biceps, Iliopsoas, Vastus Lateralis, and Semitendinosus. For each of the *n* = 45 possible trajectory combinations and pairwise comparisons we plotted their correlation coefficients, *R*_*xy*_(*τ*), spatial scatter visualization for both the raw afferents and pre-processed afferents as shown in Figure 4. All raw data correlations for the combined muscle were set at the 8-D (i.e., 4 muscles x 2 afferents) high dimensional space while the individual muscles were compared in 2-D space. All pre-processed data correlations for the combined muscle were set at the 3-D space while the individual muscles were compared in 2-D space.

Let *α* be the span or bandwidth of the detected correlations in the cluster and *β* be the total space of possible correlation values, where *α*/*β* is the percentage covered by the correlations values. Raw data correlations in the combined muscle set (Figure 4A) show a tight cluster within a 32.1% (i.e., where *α* is .646 and *β* is 2) of the full correlation range. However, that range expands to 98% (1.958/2) in the pre-processed set as more pairings move away from being less discriminable to more discriminable. This form of expansion was not only evident in the combined muscle grouping but also in the individual muscle groups (Figures 4B–E). Out of the four muscles, the Vastus Lateralis (Figure 4D) contained the most compact clustering in the raw set with the maximal expansion, spanning a minimal 23% (0.459/2) and expanding to 98% (1.96/2) in the pre-processed set. We highlight the compact-to-expansion dynamic that occurs from raw to pre-processed afferents to show the usefulness of pre-processing in giving each task more distinction and separability to enhance classification.

**FIGURE 5:**
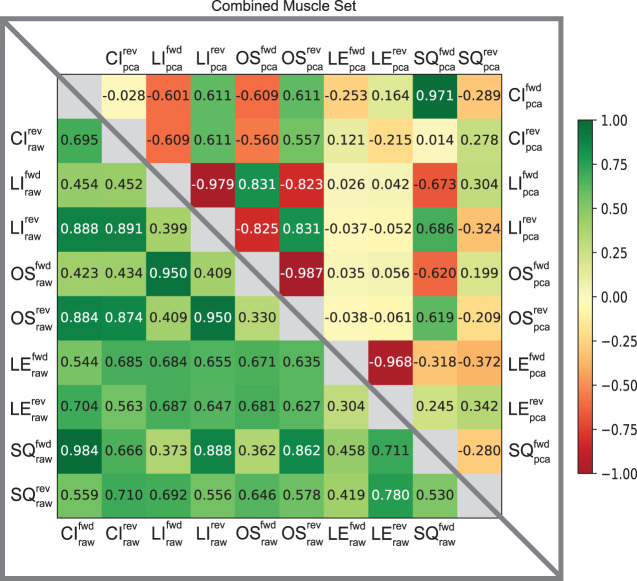
Confusion matrices of raw and pre-processed spindle afferent data for the Combined Muscle Sets. The values in each cell describe the likelihood that two movements cannot be disambiguated from each other. I.e., the more towards green, the greater predicted similarity; the more towards the red the greater the predicted difference between any two movements. Values below the diagonal in the lower triangle correspond to predictions of similarity using the *raw* Ia and II signals. Upper triangle entries represent the predicted similarity after PCA using the first principal components were used here (as in Figure 4A). We compared across all five trajectories; CI: circle, LI: line, OS: oscillatory, LE: lemniscate, SQ: square in both directions (forward *fwd* vs. reverse *rev*). This comparison is for spindle signals from all four tendons shown in Figure 1. Note that the upper triangle entries demonstrates that using PCA enables much greater discriminability among movements from spindle signals, compatible with what was found by Rongala et al. (2018) for tactile signals.

Furthermore, the usefulness of cross-correlation is additionally investigated in this study in the context of a state classifier. We find the display of confusion matrices as heat maps particularly useful here because of the ability to describe and visualize the performance of our classification model. The varied patterns of afferent correlations can be observed for all task trajectories by noting the shift in discrimination rates of the raw afferents (lower triangle) *versus* pre-processed (upper triangle) in Figure 5. The differences in discriminability vary significantly by each matrix. Our combined muscle set reports 29% discriminability among the 10 possible trajectories in the raw 8-D set and drastically increases to 82% in reduced 3-D pre-proccessed set, as shown in Figure 5. For the Anterior Biceps muscle (Supplementary Figure S1A), cross correlation reports 60% discriminability in the raw 2-D set and increases to 73% in 2-D pre-processed set. The Iliopsoas muscle (Supplementary Figure S1B) reports 66% and 73% discriminabilities, Vastus Lateralis (Supplementary Figure S1C) reports 0% and 78% discriminabilities, and Semitendinosus (Supplementary Figure S1D) reports 49% and 89% discriminabilities, for the raw and pre-proccessed set, respectively. The difference in correlation between the two groups (i.e., Raw and PCA) was determined to be statistically significant (*p* = 0.001), according to the Wilcoxon signed-rank test, for the combined muscle group and individual muscle sets.

## 4 Discussion

In this paper, we focused on using high-dimensional Ia and II proprioceptive signals from muscle spindles to enable robust estimates of limb trajectories. In particular, we tested whether the time histories of such spindle signals suffice to discriminate from among five limb movements. We find that raw multi-dimensional sensory ensembles of the muscle spindle remain within a particular range for each movement and can be discriminated. Secondly, pre-processed data shows high correlation of spatio-temporal maps between sensory and motor space. Thirdly, the correlation index revealed markers of sufficient discriminability and classification among spindle afferents. Our findings closely match with similar results from Rongala et al. (2018), where biological data on cuneate nucleus neuron recordings in adult cats were obtained and modeled to study generalizable tactile representations. Their work highlights that the cuneate nucleus forms the first interface for the sense of touch in the brain. Our results suggest that this may apply to other proprioceptive sensory afferent pathways such as muscle spindles. Triangle matrices of correlations, similar to our analysis, demonstrated how weighted learning in the cuneate nucleus resulted in decorrelated responses between neurons of the same stimulus. Essentially this means the data were less ‘confused’ with another and more discriminable. Here, we have provided an analytical approach to study and justify the nature and effects of dimensionality reduction in central or peripheral nervous system (Rongala et al., 2018; Beyeler et al., 2019) that has been of great focus in the field of biology which has recently gained greater interest.

Altogether, our findings indicate that sensory afferents from the muscle spindle can adequately supply the nervous system with features of discrimination to distinguish one task from another—but only if there are suitable forms of pre-processing or filtering to reduce the amount and dimensionality of sensory information flooding the nervous at a given time during the selection or performance of an action or task. This is also a desirable ability for dynamic models of body representations or body schemas in biological (Cisek and Kalaska, 2010; Berry and Valero-Cuevas, 2020) and neuro-robotics systems (Marjaninejad et al., 2019; Kudithipudi et al., 2022).

Moreover, the lack of centralized brains or brains with limited computational abilities in animals (Schwab and Coates, 2003; Swanson, 2004; Borrelli, 2007; Healy and Rowe, 2007; Cisek and Kalaska, 2010; Aflalo et al., 2015; Scheffer and Meinertzhagen, 2019; Pipkin, 2020) would naturally benefit from this kind of ‘edge computing’ where even minimal pre-processing of multi-muscle ensembles of spindle signals improves discriminability of limb movements (Rongala et al., 2018) and modular cortical and subcortical functions (Cisek and Kalaska, 2010; Merel et al., 2019; Li et al., 2020).

## Data Availability

The raw data supporting the conclusion of this article will be made available by the authors, without undue reservation.

## Supplementary material

SUPPLEMENTARY FIGURE S1 While in individual muscle analysis such as **(A)** Anterior Biceps, the first two principal components were selected. The same cross correlation was performed for the remaining muscles which include **(B)** Iliopsoas, **(C)** Vastus Lateralis, and **(D)** Semitendinosus.
