# Supplementary material for: Edge Computing in Nature: Minimal pre-processing of multi-muscle ensembles of spindle signals improves discriminability of limb movements
Source: Front Physiol. 2023 Jun 29;14:1183492. doi: 10.3389/fphys.2023.1183492 (PMC10345157; doi:10.3389/fphys.2023.1183492)
Supplement: Supplementary file 1 [file Image1.pdf]

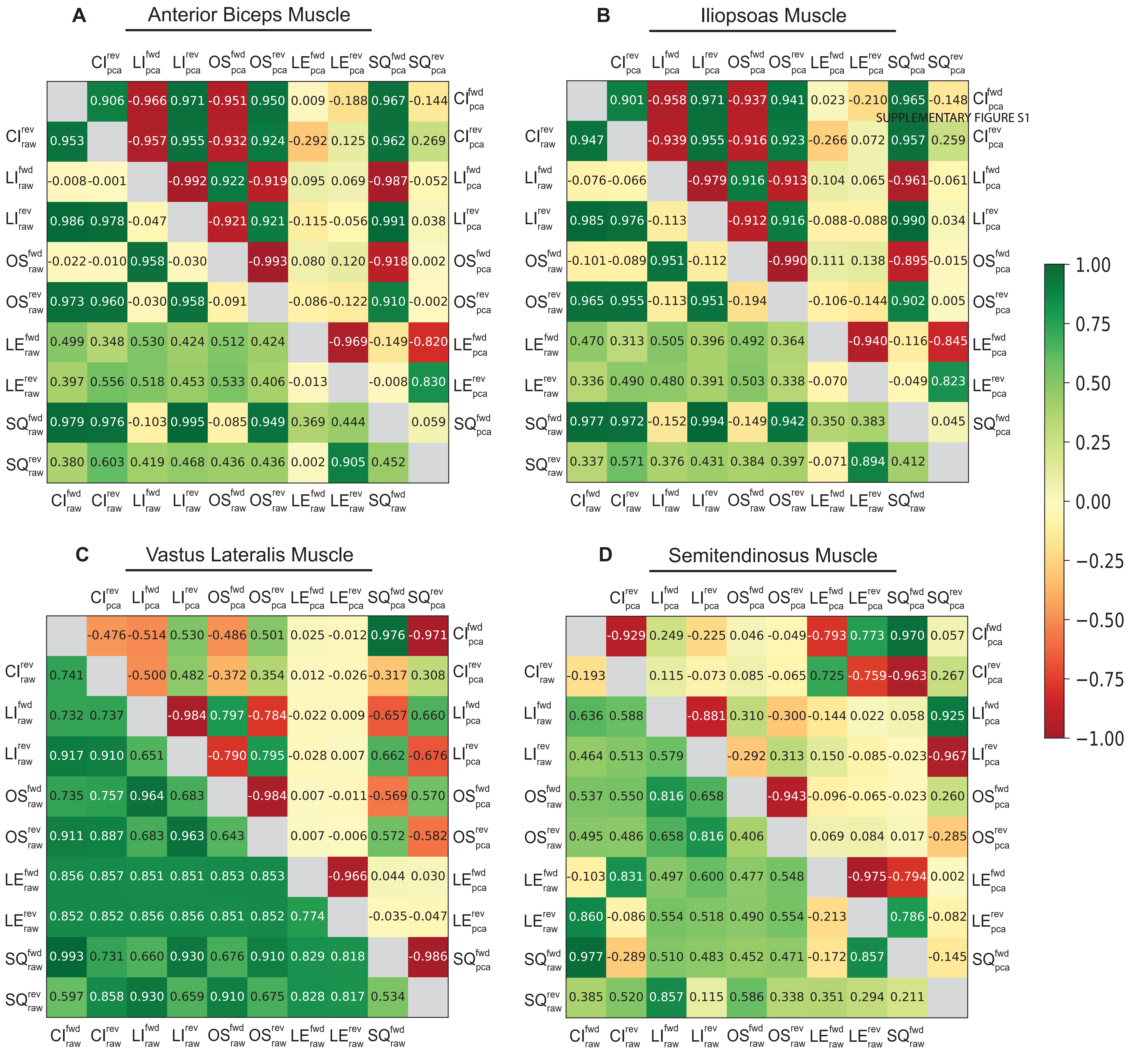

SUPPLEMENTARY FIGURE S1

While in individual muscle analysis such as (A) Anterior Biceps, the first two principal components were selected. The same cross correlation was performed for the remaining muscles which include (B) Iliopsoas, (C) Vastus Lateralis, and (D) Semitendinosus.
